# SMS-based digital health intervention in Rwanda's home-based care program for remote management of COVID-19 cases and contacts: A qualitative study of sustainability and scalability

**DOI:** 10.3389/fdgth.2022.1071790

**Published:** 2023-01-09

**Authors:** Abdulaa Babili, Sabin Nsanzimana, Edson Rwagasore, Richard T. Lester

**Affiliations:** ^1^ Faculty of Public Health and Policy, London School of Hygiene and Tropical Medicine, London, United Kingdom; ^2^ Central Administration, Rwanda Biomedical Center, Kigali, Rwanda; ^3^ Department of Public Health Surveillance and Emergency Preparedness and Response, Rwanda Biomedical Center, Kigali, Rwanda; ^4^ Faculty of Medicine, Division of Infectious Diseases, University of British Columbia, Vancouver, BC, Canada

**Keywords:** digital health, mHealth, Rwanda, East Africa, COVID-19, pandemic preparedness and response, outbreak management, implementation sceince

## Abstract

**Background:**

COVID-19 pandemic resulted in unprecedented global health challenges. Rwanda identified its first COVID-19 case on March 14, 2020 and subsequently introduced Home-Base Care (HBC) Program in August 2020 following community transmission of the virus and to alleviate logistical and financial strain on the healthcare system. Cases and contacts eligible for HBC were remotely supported by WelTel, an SMS-based mHealth intervention that was successfully implemented before for HIV epidemic in Rwanda. Enrolled cases and contacts were supported and monitored daily *via* their cell and/or mobile phones until they complete isolation/quarantine period. This study explored the rationale, perspectives, and experiences of key informants (KIs) during the implementation WelTel's mHealth tool for HBC in Rwanda.

**Methods:**

Semi-structured one-on-one virtual interviews were conducted with KIs in this qualitative study. The KIs were classified into 2 major categories: (A) Senior staff including policymakers, directors, and senior managers; (B) Technical teams including case managers, and other staff supporting the implementation of WelTel (e.g., IT staff). Interviews were audio-recorded, transcribed, and analyzed in NVivo. Thematic analysis was conducted using a hybrid approach. A topic guide was developed using the Modified Consolidated Framework for Implementation Research and feedback from local stakeholders.

**Results:**

7 KIs were interviewed. Five themes emerged following thematic analysis including: SMS-Based mHealth for Home-Isolation; Facilitators for Intervention Adoption; Barriers for Intervention Adoption; Infection prevention and control for Home-Isolation; and SMS-Based mHealth for Future Pandemics and Epidemics. Based on interviews, strong political commitment and advanced digital infrastructure were major facilitators for adopting WelTel for HBC. A major barrier to adopting WelTel was identified as technical-based issues. This was followed by local communication culture. All participates agreed on the significance of using WelTel to improve access and adherence to infection prevention and control measures, understand transmission dynamics, and inform public health decision-making regarding HBC.

**Conclusions:**

Rwanda successfully adopted WelTel for supporting and monitoring COVID-19 cases and contacts in home-isolation and the implementation was instrumental to the country's effort to manage the pandemic. Experiences and perspectives of cases and contacts enrolled into WelTel must be explored to understand the appropriateness and effectiveness of the intervention.

## 1. Introduction

On March 11, 2020, the World Health Organization (WHO) classified Coronavirus disease (COVID-19) as a pandemic (1). As of September 2022, almost 6.5 million people have died due to COVID-19 and around 612 million cases of infection have been confirmed globally (2). The pandemic urged healthcare systems to accelerate the adoption of innovative and novel solutions to address the social, economic, and health impacts of COVID-19.

Digital health interventions (DHIs) including mobile health (mHealth) are information and communication technologies that provide functionalities to address health needs and achieve healthcare system's objectives (3). mHealth, is defined as the use of mobile technologies in support of health needs (4). The rapid deployment and scale-up of DHIs during the pandemic ensured the continuity of healthcare services while adhering to public health measures and containing the spread of COVID-19 (5, 6). In the last decade, the incorporation of DHIs within outbreak response systems and surge capacities has expanded significantly (7). DHIs are currently used for contact tracing, symptoms reporting and tracking, targeted public health messaging, and virtual clinical consults (7). The mushrooming of app-based mHealth interventions can be advantageous in contexts with large access to smartphones, high internet coverage, and high digital literacy (8). Otherwise, they can exasperate health inequalities and exclude vulnerable groups due to access (9). Accordingly, program planners need to critically consider logistical aspects such as local system readiness and regulatory frameworks; local culture and willingness to use DHIs; and DHIs specific ethics such as data privacy when implementing DHIs (5, 10). Furthermore, the use of DHIs could contribute to increasing health inequalities due to low digital literacy among vulnerable populations, and the digital divide on a national level created by suboptimal access to digital devices and the internet (10–12). Despite the projected increase in the number of mobile-internet subscribers in Sub-Saharan Africa (SSA), the region still ranks the lowest with 270 million subscribers (26% of the total global subscribes) (13, 14). This gap is larger among rural populations as they are 37% less likely to use mobile internet compared to urban populations (13, 14).

In the absence of treatments and vaccines for COVID-19, many countries implemented non-pharmacological interventions such as institutional-based isolation and contact tracing to control the spread of the virus (15, 16). While institutional-based isolation can reduce community transmission of COVID-19 compared to home-based isolation of COVID-19 cases and contacts, it presents an unsustainable solution particularly in Low- and Middle- income countries (LMICs) (15, 17–19). A scoping review found that home-based isolation was associated with reduced risk of stigmatization and provided safe space for patients to express their needs (17). Additionally, home-based isolation allowed the decongestion of health facilities (17). Some challenges can impact the effectiveness of home-based isolation to contain the transmission of COVID-19. Cases in home-isolation are more likely to infect household members (15, 17, 20). Poor knowledge and suboptimal practice of infection prevention and control measures among isolating cases was associated with high household secondary attack rates (17, 20).

**Figure 1:**
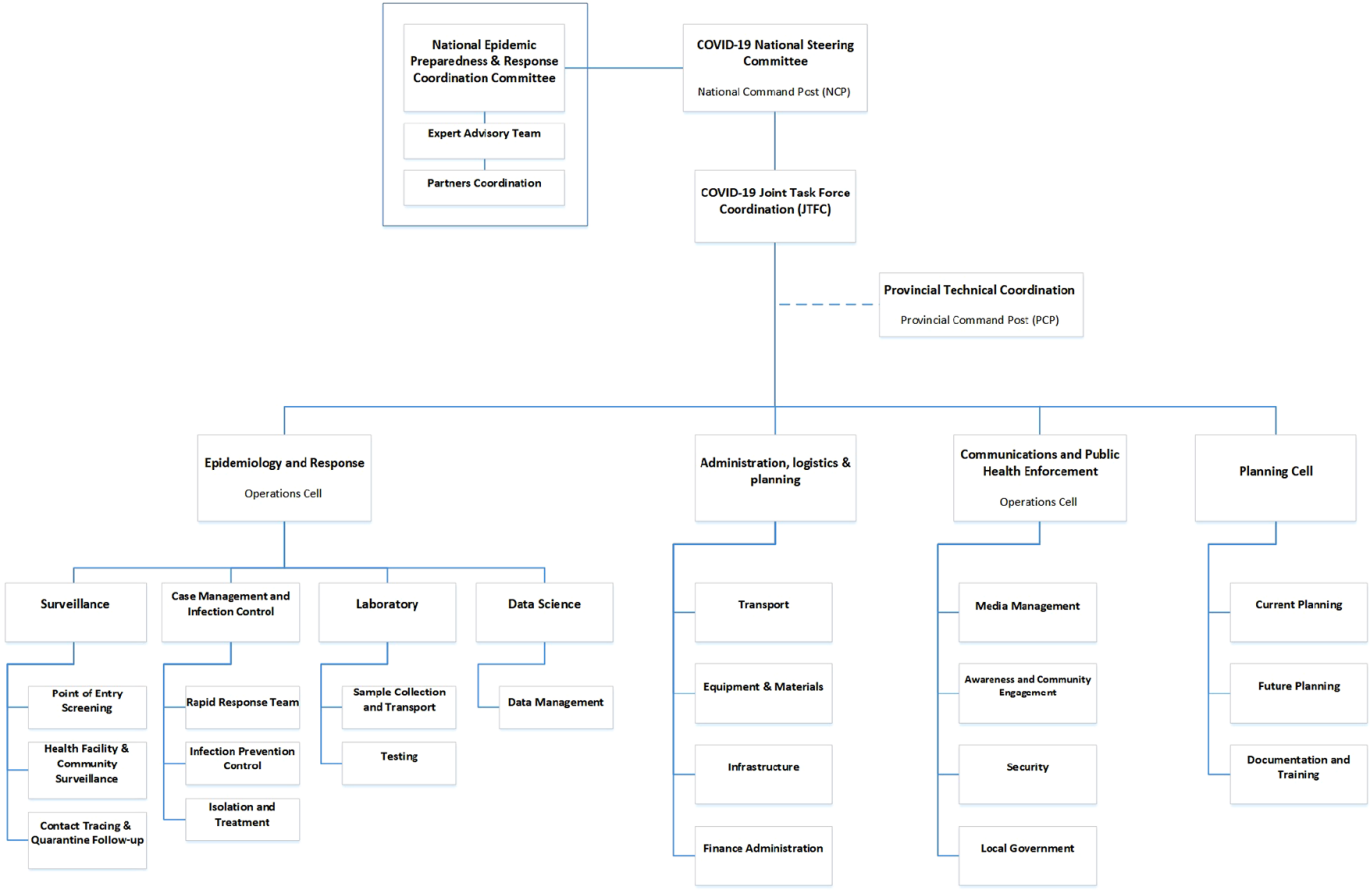
Structure Diagram of the COVID-19 Joint Task Force in Rwanda: This figure was adopted from Karim.24.

Rwanda is an East African country with a total population of 13,705,393 as of 2022 (21). The country is made up of 5 provinces: Northern province, Eastern province, Southern province, Western province, and Kigali City (22). Each province is further broken down into districts. Kigali City province, the most densely populated province, consists of 3 major districts including Gasabo, Kicukiro and, Nyarugenge (21, 23). Rwanda is recognized as a leader in healthcare and an early adopter of DHIs within the East African region (24). It was among the first African countries to prepare for the fight against COVID-19 starting as early as January 2020 (25, 26). On March 03, 2020, the COVID-19 Joint Task Force (JTF) committee was established to provide strategic guidance, coordinate and manage the implementation of a preparedness and response plan including day-to-day operational and logistical support (Figure 1) (25, 26). The committee consists of subject matter experts from numerous ministries and local academic institutions to enable a multi-sectorial approach to the management of the pandemic nationally. Upon identification of the first COVID-19 cases on March 14, 2020, Rwanda Biomedical Center (RBC), the national health implementation agency, introduced testing, contact tracing, and facility-based isolation and quarantine (26, 27). Rwanda's containment measures were among the stringiest in SSA (28). During the first wave of COVID-19 between March and July 2020 Rwanda's isolation facilities experienced an overload due to rising number of cases and limited human capital resources to support the surge capacity. To reduce the increasing costs and alleviate strain on isolation facilities, Rwanda introduced the Home-Based Care (HBC) program in August 2020 during its second wave (18, 29, 30). According to the Minister of Health, Dr. Ngamije, “The initiative has been effective …  with home-based care there are expenditures which will be avoided such as cost of accommodation at quarantine sites and feeding.” (19, P. 1). HBC was adopted because 70% of all cases were mild or asymptotic and did not require facility isolation (18). Furthermore, Rwanda saw a sharp increase in incidence and mortality rates of COVID-19 due to the emergence of variants of concern particularly Beta and Delta variants since June 2021 (31, 32). This increase was the highest in Kigali City province (33, 34). As of September 2022, the number of confirmed COVID-19 cases was 132,454 and the number of deaths was 1,466 (35).

**Figure 2:**
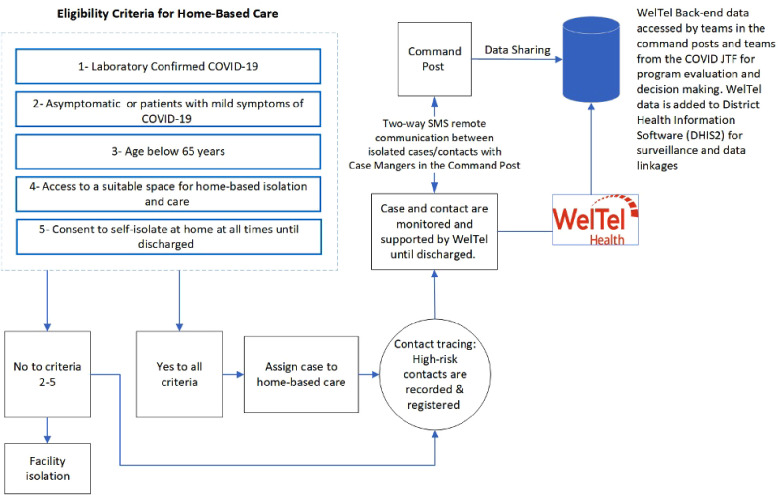
Algorithm for Eligibility to Participate in Home-Based Care: Cases and contacts who meet the eligibility for Home-Based Care will be assigned to WelTel for remote monitoring and support.

To be eligible for HBC, patients had to meet a set of criteria (Figure 2). Cases and contacts in HBC were enrolled into WelTel digital health platform using their phones (30, 36). WelTel is a digital health platform that facilities two-way communication between healthcare providers (HCPs) and patients (referred in this study as cases and contacts) (37). It is a web-based platform that utilizes Short Message Service (SMS) as its core functionally while allowing for other modes of communication such as video calls (37, 38). WelTel sends automated SMS check-in messages to registered patients asking, “How are you?”. The messages are sent based on a specific frequency (e.g., daily/weekly/monthly). Patients' responses are received and triaged by the platform for HCPs to follow-up (38). Patients can respond to check-in messages at any time. Other functionalities like contact tracing, reminders, and data export can be accessed when using the platform (3). The “How are you?” SMS message is based on WelTel's “Ask, don’t tell’ approach, where patients have full control over the expression of their health needs rather than being asked or reminded of specific tasks (39). In response to COVID-19, WelTel was optimized for case management and contact tracing (3). A report from Johns Hopkins University assessed the use of WelTel for COVID-19 case management and contact tracing in LMICs (3). The report defined criteria necessary for a successful deployment of DHIs including, scalability, interoperability, and messaging capability. The report concluded that WelTel meets some of these criteria including usability and messaging capability but falls short in other criteria like interoperability (3). RBC adopted WelTel mHealth intervention for real-time remote monitoring and support of COVID-19 cases and contacts in HBC in Kigali City province (Supplementary Figure S1) (30, 36). The platform sends daily check-in messages for 14 days. This allows for self-reporting of symptoms or concerns, two-way SMS communication between cases/contacts and case managers, and proactive engagement (30, 36). Lastly, the platform centralizes data and functionalities for easy access (3, 37). Cases and contacts in Rwanda interact with teams in various command posts across the country for support (40). Command posts are centers with teams that support interaction and engagement with cases and contacts in HBC and communicate information back the designated JTFs structures to evaluate public health interventions and plan responses accordingly. Case and contacts are expected to contact the teams at the command posts for support. Teams at the command post have access to the WelTel's back-end to manage cases and contacts interactions and data exports. Even though the platform has been tested in different disease and geographical contexts, the application of WelTel for HBC and COVID-19 is novel and further scientific evaluation is necessary to ascertain feasibility, effectiveness, and impact. The adoption of WelTel in Rwanda offered a real-life example for countries with similar geographical, cultural, and economic characteristics. As of June 2020, almost 1 billion SMS messages have been sent in Rwanda (41). Additionally, texting is the most common type of activity among Rwandan mobile users (41). The use of SMS-based mHealth interventions can provide a comparative advantage to using traditional phone calls or smartphone apps. They offer an inclusive and cost-effective approach to transmit information at a faster rate to many people (42–44). Finally, WelTel was adopted in Rwanda in 2017 for 5 years to support HIV patients and has been since serving many patients remotely in Kigali Province (45).

**Figure 3:**
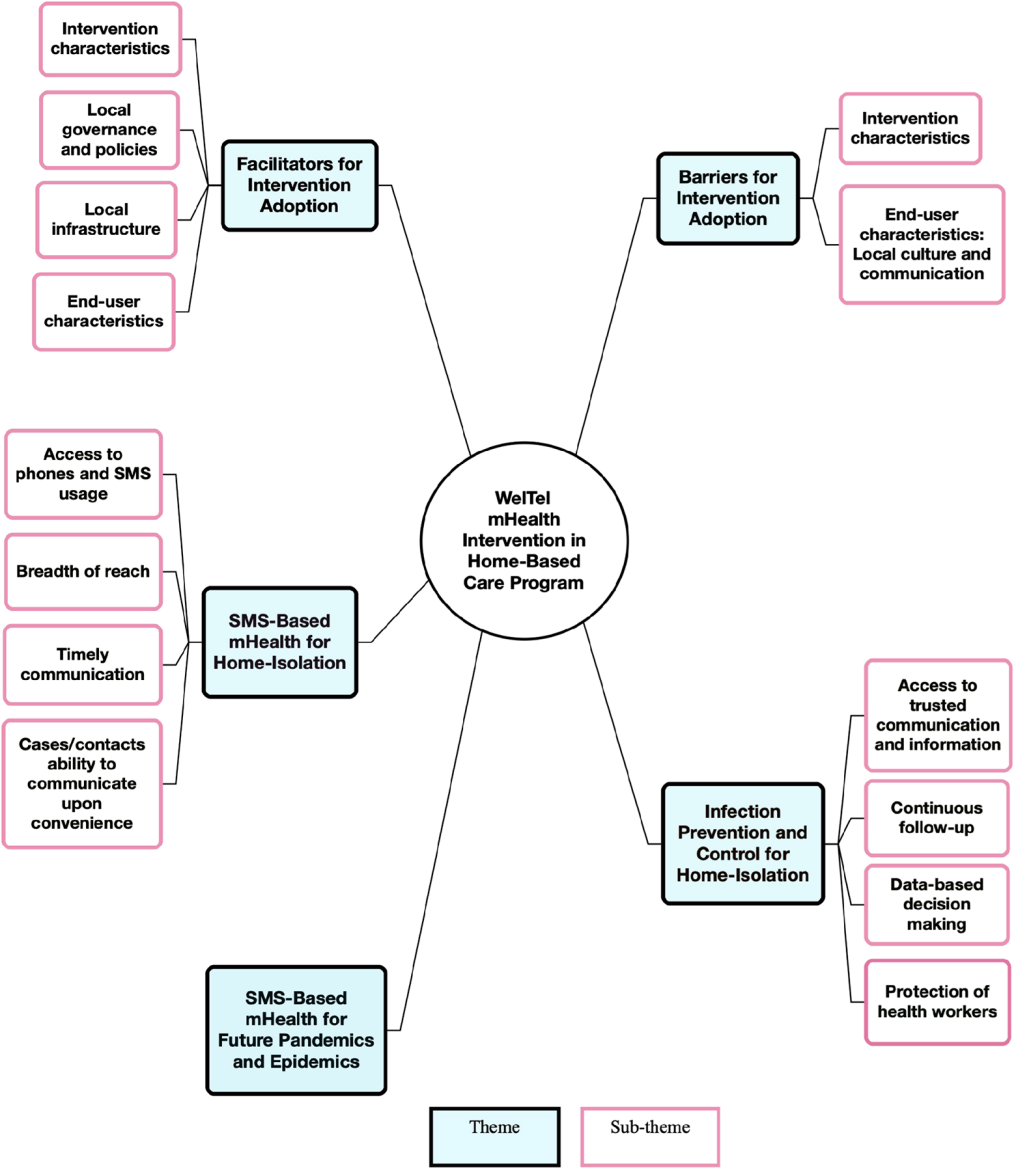
Thematic map: key themes and sub-themes.

The aim of this study was to evaluate the rationale, experiences, and perspectives of key informants (KIs) in Rwanda towards implementing WelTel SMS-based mHealth intervention in Rwanda's HBC program for COVID-19 cases and contacts management, and to examine factors that could impact its adoption and scalability. The study also sought to examine and describe barriers and facilitators to the adoption and scale-up of WelTel as part of HBC, and to explore the overall impact of the WelTel intervention on the HBC program through the perspective of KIs.

### 2. Materials and method

### 2.1. Study design

An exploratory research approach was adopted in this study. The qualitative design allowed for the examination of participants' rationale, perceptions, and experiences, and to gain an in-depth understanding of this topic given the limited research available. The Standards for Reporting Qualitative Research (SRQR) was adopted to guide the structuring of this report and to enhance transparency in the reporting of this qualitative study (46). Due to COVID-19, it was not feasible to travel and conduct the study in Rwanda; the study was thus conducted virtually using Zoom (47). Virtual semi-structured one-on-one interviews were conducted with KIs. The semi-structured nature of the interviews allowed for both guided and in-depth discussions; the open-ended nature of the interviews allowed the interviewees to bring forward novel topics.

### 2.2. Topic guide and framework

A standardized topic guide was developed and used to guide the interview sessions, though, different probing questions were asked for further clarification and/or elaboration from participants. The topic guide used for this study was reviewed by stakeholders at RBC to provide local perspective. Feedback was integrated into the final document. The Modified Consolidated Framework for Implementation Research (mCFIR) was used to guide the development of some sections in topic guide (38, 48, 49). The mCFIR is a tool based on the original CFIR framework and modified by the mHealth Research Group to evaluate specific aspects of DHIs implementation and was successfully applied in implementation science studies (38, 48–51). The CFIR is a meta-theoretical framework constructed from 19 theories and frameworks which offers theoretical rigor and comprehensiveness in evaluating the implementation of interventions within a specific context (38, 52). The CFIR and the mCFIR have been validated in LMICs (38, 48, 53). The mCFIR can therefore provide the ability to examine a wide range of determinants related to adoption of mHealth interventions within a specific context, and guide future implementations and scale-up (38, 48). The mCFIR tool consists of 25 constructs under 5 major domains (Supplementary Table S2). The domains are well-encompassing of all aspects and stakeholders involved in the implementation.

### 2.3. Sampling and recruitment

Participants were selected purposively based on the following criteria: (1) participants working directly with WelTel to manage cases and contacts in HBC; (2) participants took part in devising the plan to implement WelTel and/or HBC and exposed to day-to-day operations of WelTel and case management; (3) participants are comfortable to speak English. Purposive sampling ensured maximum variations in sampling. A total of 12 KIs were invited for this study. An invitation email was sent to all identified participants. Participants received a Participant Information Sheet before consenting to the participation in the study in the email invitation. After receiving the email, participants were asked to respond with whether they are interested in joining the study. Participants were asked to provide their availability to set up the interview session. Oral consent was taken at the start of the interview session.

KIs were classified into 2 major categories: (A) Senior staff including policymakers, directors, and senior managers in RBC; (B) Technical teams including case managers, and other staff supporting the implementation of WelTel (e.g., IT staff). The objective behind this classification was to capture information from senior-level personnel and frontline personnel and to obtain different perspectives and experiences to add breadth to the results. The study attempted to sample an equal number of participants from each category.

The study aimed to interview 12 participants. Due to the critical position of the KIs in the national response to COVID-19, the ever-changing nature of the pandemic, and the limited time available to conduct this study, 7 participants were interviewed. Additionally, Rwanda was in its third COVID-19 wave and KIs identified in this study were overwhelmed with the emergency response. However, the KIs interviewed in this study were instrumental (core team) to the planning and implementation on the mHealth component of the HBC program in Rwanda. Accordingly, the interviews generated relatively adequate data to address the aim of the study and improve internal validity.

### 2.4. Data analysis

Thematic analysis was conducted using a hybrid approach of deductive and inductive analysis for code and theme identification and generation (54–56). This approach provided rigor to addressing the aim of the study by allowing the incorporation of the mCIFR domains and questions from the topic guide in the deductive analysis. Concurrently, it allowed for novel themes to emerge from the data using inductive analysis. The “Six Steps process to thematic analysis” was adopted from Baruna et al. to ensure a systemic thematic analysis (55, 57).

Interviews were audio-recorded, anonymized, and transcribed verbatim (47). Transcripts were then uploaded on NVivo software for data management and analysis (58). To ensure accuracy, revision of transcripts occurred while listening to audio recording. This stage also allowed for familiarization with data and the generation of initial codes. Codes were merged into sub-themes and themes. Revising the generated sub-themes and themes occurred by reviewing all the codes relevant to each theme and ensuring that they fit within the theme.

Data analysis started after the first interview and the process was iterative. A pre-coding mind map (Supplementary Figure S2) was created *via* NVivo as a brainstorming tool before data collection and analysis. This mind map represented the coding framework of this study. The development of the mind map was iterative, and adjustments were made throughout the interviewing process. Finally, to protect participant's anonymity and confidentiality, the study anonymized quotes and applied pseudonym names.

Given that qualitative data is generated through researcher-participant interaction, this study considered reflexivity as a strategy to identify and control for bias in data generation and interpretation. The researcher's personal experiences, professional affiliation, and values can impact the way questions are developed and asked in qualitative research, as well as how data is interpreted and presented. Thus, continuous evaluation of positionality and critical self-appraisal is essential in qualitative research (59). Accordingly, a declaration of positionality was provided in the discussion.

### 2.5. Ethics

This study was reviewed and approved by the Research Ethics Board committee of the London School of Hygiene and Tropical Medicine. Questions about COVID-19 might trigger personal experiences of grief. Therefore, there was a very small risk of emotional distress due to KIs involvement in the response to the COVID-19 pandemic. This was mitigated by verbal assurance to not answer any questions perceived distressing and providing contact information of local mental health services offered to people affected by the pandemic.

## 3. Results

### 3.1. Summary of participants

Seven KIs were interviewed between July and September 2021 (Table 1). Of the 12 invited to participate in the study, 5 rejected participation due to time constraints. Three participants were from category A – senior staff, and four were from category B – technical team. Of the four participants from category B, one was an IT staff. All participates are working in RBC, and participants from category A are all members of the JTF. The duration of the interviews ranged between 20 and 70 minutes. Some interviews were shorter in length due to participants' limited time. One interview was carried over 2 different times due to poor internet connection. Participants from category A were heterogeneous in terms of their positions.

**Table 1:** Summary of participants.

Participant pseudonym name^a^—(category A or B)	Position	Major tasks related to the position
Josh-(A)	Chief Data Scientist	Directing the design and implementation of data science and information technology solutions for COVD-19.
Marie-(A)	Division Director	Directing the design and implementation of infection, prevention and control protocols for highly pathogenic infectious diseases.
Innocent-(A)	Director—Case Management & Infection Prevention and Control (IPC)	In charge of case management and IPC teams for both home- and facility-based isolation.
Vincent-(B)	Program Manager in a Non-Governmental Organization (NGO) & Project Consultant at RBC	Monitoring and evaluating IT infrastructure and related tasks for the mHealth interventions in RBC including WelTel
Christine-(B)	Case manager-HBC	A direct user of WelTel and communicates directly with cases and contacts in HBC. Command Post
Eric-(B)	Case manager-HBC	^b^
Jacques-(B)	Case manager-HBC	^b^

Note: RBC, Rwanda Biomedical Center; HBC, Home-based Care; JTF, COVID-19 Joint Task Force; IPC, Infection Prevention and Control.

^a^ Participants were assigned pseudonyms names to ensure confidentiality.

^b^ Similar to the above section.

### 3.2. Qualitative findings

5 themes were identified through analysis (Figure 3) including 1-SMS-Based mHealth for Home-Isolation; 2-Facilitators for Intervention Adoption; 3-Barriers for Intervention Adoption; 4-Infection Prevention and Control for Home-Isolation; and 5-SMS-Based mHealth for Future Pandemics and Epidemics. Themes 2 and 3 have been deductively analyzed based on the mCFIR framework and topic guide. Themes 1,4 and 5 inductively emerged from the interviews.

#### 3.1.1. SMS-Based mHealth for home-based isolation

Almost all participants shared similar rationale and perspectives for selecting WelTel for HBC. This theme emerged because participants discussed the rationale for the HBC program. Participants stated that HBC was adopted because 1-majority of cases are mild or asymptomatic, and 2-to reduce strain on the healthcare system. Additionally, it seemed that cases favored home-isolation compared to facility-isolation. Innocent, Director—Case Management & Infection Prevention and Control, said:

“*With [home-based] care you have gained the satisfaction from people. Like, ‘I (case) I don't want to go to that facility with people that I don't know, with eyes all around me, I feel so pressured. I feel so stressed”*.

Four reasons have been identified as major factors for adapting the WelTel platform (Figure 3).

Firstly, all participants mentioned mobile phone access and SMS usage as factors for selecting WelTel. Participants emphasized that the Rwandan population, both in urban and rural areas have adequate access to at least basic mobile phones and are used to SMS. Josh, Chief Data Scientist at RBC, said:

“*At least every household has a cell phone, [therefore it] was simpler and easier to implement WelTel, because you are sure that at least in every household you find one phone used to send them SMS, to get responses, and to [follow-up] patients at home.”*

When asked about other solutions that could've been implemented instead of WelTel, 6 participants alluded to the use of app-based DHIs. However, there was consensus that such interventions can exclude patients with no access to smartphones. Christine, a case manager, said:

“*[The] percentage of people in Rwanda that use smartphones and have access to internet is still low. But access to regular phones not smartphones is high, to an extent that in every home at least you will find one holding a phone, may not be a smartphone, it may be regular phone”*

It is essential to reach many people in a timely and reliable manner. According to Josh and Innocent, this was a goal set by the JTF during the planning of HBC to adopt an intervention that allows for instant communication with cases and contacts in home-isolation, while enabling them to reply in a convenient time.

“*We considered accessing more patients at a more convenient time.” [Josh]*

Six participants discussed WelTel's ability to reach many cases promptly. They shared the perception that for a DHI to be scalable in a pandemic, it must be efficient and effective at reaching a large number of cases on time. Participants from category B have commended the platform's ability to follow-up with many people on time. Jacques, a case manager, said:

“*Using SMS is so easy and [quick] for people to receive the information you want to communicate with them”*

KIs recognized the importance of such ability especially when case numbers started to increase, and in-person follow-up wasn't feasible.

“*Because the numbers are high, you find in a day close to 1000 cases. And so, no one will be able to go to every door knocking, to check on this patient. But if I have a system that can cover more than 500 patients in a day, following them just in one location, it becomes more friendly, cost-effective.” [Christine]*

Josh mentioned the ability of the platform to send bulk-SMS to a large number of people simultaneously, giving an example of WelTel's ability to reach large number of cases.

Two-way messaging and on-demand communication were highlighted as important factors to selecting WelTel compared to other DHIs that fall short in this regard. They were considered important aspects for optimal and safe remote case management and support. Josh compared WelTel's ability of two-way SMS communication to a previously implemented DHI.

“*We have used an SMS system. But the experience was different from WelTel. The difference with WelTel is that it’s [two-way] and the previous system was only [one-way] where you send SMS and don't expect feedback from the patient. It's like you're informing but you don't get feedback. But through WelTel you send SMS, and you can receive SMS from the patient, or someone you're following up, which makes a big difference because it helps to react on time, act basing on the feedback. Which makes WelTel more advantages compared to the system that we had before.”*

Christine, Eric, and Innocent compared using phone calls as a tool for remote monitoring of cases in home-isolation to using two-way SMS.

“*Compared to phone calls, phone calls are annoying sometimes. Some patients say that we are disturbing them….When you ask the patient using phone call, sometimes they don't have a quick response or any other information. But when they are writing a message, they take time so they can write how they're feeling and any changes.” [Eric]*

All agreed that phone calls can limit the ability of cases and contacts to express their needs while on the call, especially if the call is placed at an inconvenient time.

This shared experience illustrates the added benefit of using SMS-based systems to follow-up with cases in home-isolation.

#### 3.1.2. Facilitators for intervention adoption

Facilitators are defined in this study, as factors that enable the implementation and scale-up of DHIs within a specific disease and geographic context. The theme is divided into 4 sub-themes including intervention characteristics, local governance and policies, local infrastructure, end-user characteristics.

##### 3.1.2.1. Intervention characteristics

Technical features and cost-related aspects of the tool that enabled its adoption for HBC are outlined. Participants emphasized the importance of data centralization in enhancing the ability to monitor cases and contacts through improved efficiency and consistency in capturing data. Innocent and 2 case managers suggested that switching from paper-based systems to electronic-systems like WelTel for remote case management was an advantage.

“*It saves time. It's precise and it leads to higher efficiency. You have your data at hand and consistency when collected, as opposed to have a pile of papers …. So WelTel in the management of [home-based] care saved us from having piles of files on cases that are not easy to retrieve, and not completely captured.” [Innocent]*

Data centralization was linked to the multimodality of the platform. The two features combined resulted in streamlining case managers' work and enhancing the ability to follow cases and contacts through timely access to data and usage of available features.

“*We wanted a tool that provides many options and futures, that would give us much information about what is going on with the patient at home” [Josh]*

Furthermore, data centralizations aided the decision-making process regarding case management and HBC.

“*If you have one platform, which can enable you to chat with a patient, and use video function. Then you're getting all those data in one platform. It made it easier for the analysts to take a decision based on the information from different features being used.” [Josh]*

All participants shared the importance of a user-friendly interface for daily usage and training.

“*It's user friendly, after using it like one week, you know how the system works” [Vincent]*

Cost was broken into two types: 1-cost incurred by implementation agency; 2-cost incurred by cases and contacts.

Cost associated with implementing WelTel for HBC was perceived as low. According to participants from category A, this is because WelTel has already been established in the country for HIV virtual care. Consequently, logistical, and technical costs to adopt WelTel were not perceived as a barrier. Marie, Division Director, said:

“*We did not invest so much in the system when it came to COVID. Because it had already been established, [it] was already in place. We just only had to tweak a few things.”*

Following theme 1 which described the breadth of reach, participants shared the perspective that digitized systems can cut costs by reaching many people from a single platform. Vincent, Program Manager in a Non-Governmental Organization (NGO) & Project Consultant at RBC, said:

“*Purpose of a digital system is to ease work, get results, speed up processes and cut costs of doing things.”*

The cost incurred by cases and contacts when enrolled into WelTel was interpreted based on discussions with participants. Cases and contacts had no out-of-pocket expenses when it came to using SMS. Cases and contacts appeared to be aware that SMS sent from RBC have no costs associated with receiving and responding to them. This is due to the reverse-billing of SMS costs to RBC rather than the sender (cases and contact).

“*It's a [toll-free] system. They know, once they get the messages they see RBC, they know that it's RBC following, and replying to us doesn’t cost them.” [Christine]*

Finally, Eric and Christine briefly touched on data security aspects of the platform, stating the advantage of automatic timed log out once the platform is not in use. Otherwise, no further discussion of larger system-based privacy and security features has been brought up by KIs.

##### 3.1.2.2. Local governance and policies

Rwanda was pioneering a digitization process across all governmental sectors. This was seen by most participants as an important facilitator to implementing WelTel. According to participants, policies for incorporating DHIs within the healthcare system accelerated the willingness to adopt WelTel for HBC. There seemed to be a commitment from the government to invest in DHIs, especially during COVID which saw the accelerated adoption of DHIs.

“*We happen to have leadership that is very supportive in terms of digitalization. There were many actions to digitalization before COVID in different areas. So, digitalization of health response is something very [highly] supported, and we had encouragement to move towards that.” [Innocent]*

“*With COVID, they [accelerated] the transition plan. Because we wanted to make sure that 100% of COVID services are digitized in Rwanda.” [Josh]*

This commitment was evident when the Head of State visited one of the case management units in command posts and stated:

“*Anything that you need and is available and you don't get it, please let me know. Because I want you to feel like you should access anything that you have seen or heard is available for better response and embracing digital tools” [Innocent]*

##### 3.1.2.3. Local infrastructure

Local digital infrastructure was a forefront facilitator to the implementation of WelTel according to most participants. Participants agreed that Rwanda's digital infrastructure is ready to host DHIs. This is based on high internet coverage within health facilities and the increase in the number of health facilities across the country.

“*The coverage of network is around 85% all over the country for health sector. You can go to southern, northern, eastern, western states and you find the infrastructure, the network coverage.” [Vincent]*

“*Rwanda has this national data center. And there was much work being done to improve the capacity of servers…All the health services, and not only health services, but most of the government services are centralized to the national data center.” [Josh]*

Rwanda seemed to introduce a National Data Center to centralize national-level data and allow for optimal incorporation of DHIs in the country.

##### 3.1.2.4. End-user characteristics

The analysis identified how participants welcomed the intervention, their training and confidence to use the platform, and their perception of how this tool can target the wellbeing of cases and contacts. All participants welcomed the use of WelTel and seemed to share the enthusiasm of local policies in the adoption of DHIs.

“*I believe the future governments and the world should really consider digitizing health system” [Eric]*

Christine linked using DHIs like WelTel to a healthier society.

“*Digitizing healthcare would be a very great step towards having a [healthier] society.”*

All staff seemed to be well-trained and confident in using WelTel.

“*So, I could say the training was sufficient.” [Christine]*

There was expressed appreciation for the evolving nature of the tool, and the team's ability to keep end-users informed and trained through continuous sessions throughout the implementation period.

#### 3.1.3. Barriers for intervention adoption

Barriers, in the context of this study, are factors that challenge the implementation and scale-up of DHIs within a specific disease and geographic context. The major barriers identified are broken into 2 major sub-themes, intervention characteristics, and end-user characteristics.

##### 3.1.3.1. Intervention characteristics

The system's technical issues and poor system interoperability were identified as barriers to the adoption and scale-up of WelTel for HBC.

Rwanda has a well-established system to evaluate new technologies aimed to be implemented in the country. Even if such technologies are needed and useful, they must pass through different institutional bodies and regulatory and technical criteria for them to be implemented across the country. If a novel DHI does not meet some of the criteria, the implementation process could be hindered. One of these criteria is system interoperability. The goal of system interoperability is to enable systems to share data and save resources. Because WelTel is not a standalone system for the HBC program as mentioned by Josh, the platform must be interoperable with other systems. When asked about barriers to adopting WelTel nationally, one participant presented the fact that the WelTel system's ability to integrate and share data electronically with other systems is not fully developed. And this could have possibly hindered its adoption across all provinces.

“*In Rwanda a system needs to go through different bodies to be approved. And those bodies need to be convinced on the new platform, and there are questions that seemed to be asked even if the tool is very useful and very needed. And one of the questions is about interoperability. This was the challenge for WelTel.” [Vicnent]*

Some features of the platform seemed to create inefficacies for case managers. Manual inputting of case information into the system was time-consuming specifically on days where the number of cases and contacts is large.

“*If you have more cases in on a particular day, because the way WelTel works, you have to input people in the system manually. So, on a day, you have 50 and you have five staff, it's manageable. But if one day you have 5000 cases, then you know that's going to be a problem because you're not going to input everyone… . So, not all positive cases have been captured in WelTel, because of the load.” [Innocent]*

Because the platform depends on the internet to function from the provider side, in some sites with low internet connectivity WelTel was non-functional. Eric the case manager suggested an offline option.

“*It requires the internet to be installed for us and to connect with cases. Maybe it could be easier if it's used without internet, wherever you are.”*

Three participants highlighted the inability of the platform to conduct phone calls, which is a mode of communication sometimes employed by the HBC program. Even though participants were aware that WelTel can accommodate video calls, they emphasized the need to conduct phone calls as it doesn’t require cases and contacts to have smartphones. The importance of the calling feature is critical in situations where COVID-19 cases and contacts do not respond to messages and prompts.

“*So, the response rate was low that it required the response staff on the other side, to have to call outside of the WelTel platform to get to gauge a patient status or a contact status.” – [Marie]*

During the earlier stages of the program, only cases and contacts with a specific telecommunication provider were able to be enrolled into WelTel, which at some point meant that many cases and contacts were excluded.

“*At the beginning of the program since we had to register patients by their phone numbers to contact them. Given that Rwanda has several telecommunication companies, it only seems to capture certain numbers from a given telecommunication company, we were not able to enroll all eligible patients.” [Marie]*

Vincent raised the issue of case manages registration on the platform, which required newly enrolled case managers to create a new password through a link sent in an email invitation once enrolled into the platform. The ability to use this link expires in a short time, which meant more work for those responsible for enrolling case managers.

Finally, participants mentioned that during a certain period they noticed that they stopped receiving messages from enrolled cases and contacts for two days. When they attempted to troubleshoot the problem, they noticed that the system sent messages in a different language. Even though this has been immediately resolved, yet, in situations where many individuals are registered into a system and where time is essential, such glitches can have a considerable negative impact on the public health response to the pandemic.

##### 3.1.3.2. End-user characteristics: local culture and communication

The analysis identified cases and contacts' acceptance of WelTel through the lens of the KIs. Although this study did not explore cases and contacts perception and experiences of WelTel for HBC, KIs focused on the barrier resulting in non-responders, where the non-response rate was around 25%–30%, and some attributed it to the communication culture in Rwanda.

“*But that's the challenge because not everyone will reply…That's a challenge [when] following up with them.” [Christine]*

*“it's a good innovation, but depending on the setting, the cultural bit of the countries, I would say not everyone is responsive to messages… … . Rwandans do not always interact via texts.” [Marie]*

One participant directly cited an aversion to SMS communications to all non-SMS responders, saying they would potentially respond to phone calls.

#### 3.1.4. Infection prevention and control for home-isolation

The analysis identified the role of WelTel in facilitating safe and effective home-isolation. The central ideas in this theme include access to trusted communication and information, continuous follow-up, data-driven decision-making, and protection of health workers.

All participants shared the importance of providing cases and contacts with access to trusted communication and information through WelTel. Based on KIs, cases and contacts are satisfied with WelTel service because they can obtain reliable and “real information” on time from the HBC team. Therefore, case managers were able to use SMS-based two-way communication to convey information about COVID-19 and infection prevention and control measures at home and to answer questions promptly.

“*We receive so much, so much, infection prevention questions. Because we get those questions almost daily, patients who are trying to find out more ways how they can [interact safely] with people in household, what they should eat, and what they should do.” [Christine]*

Most notably, one participant identified the benefit of providing information to cases over SMS as it can be checked at later times by cases for reference.

“*We give them information on how to use protection methods and to stop the spread to the entire family… . So, using this system we are able to write that information so that patients can always read them and master them.” [Eric]*

It appears that some cases who have been followed *via* WelTel continued to interact with the HBC team to ask questions about COVID-19 even after they no longer received the check-in messages. An added benefit was an instance where a previously inactivated case informed the team about neighbors who are unwell and not aware of HBC and WelTel. Participants considered this a positive indicator for the success of HBC and WeTel.

“*Even after they get inactive, they keep writing messages giving us information they have, ask for information about COVID, and maybe other patients from their neighbourhood who have a problem who doesn't know about this system.” [Eric]*

Daily follow-up provided two major advantages. First, it captured data about cases and contacts on daily basis. This allowed for understanding the course of the disease, detecting new symptoms during the period of home-isolation, identifying the magnitude of secondary attack rates at home, and supporting decision making about HBC.

“*We also have been able to, to pretty much have an understanding of patient's illness throughout follow-up period” [Innocent]*

*“The other thing that worked well was we were able to tell the magnitude of positives, we were able to capture all the information that we need. See from that system, we were able to know how many patients we have that are in [home-based] care that develop mild symptoms, we also can know to what extent their contacts test positive so we can determine the positivity rate of the contacts.” [Marie]*

*“It can impact the attack rate because from the data we capture from the WelTel platform, we get to analyze the attack rate of the patients and advise going forward on what to do given at what level the attack rate is. From the analysis we get to provide some [evidence-based] findings that can be used to inform public health measures going forward.” [Marie]*

Second, daily follow-up and access to trusted communication and information positively impacted the mental wellbeing of cases and contacts in home-isolation. Innocent argued that it made cases and contacts feel safer and cared for.

“*Patients also enjoy it because they feel like we have not abandoned them”*

Finally, most participants agreed that using WelTel potentially protected contact tracers and health workers who are otherwise must interact in-person with positive cases and thus increasing their risk of infection with COVID-19. This is the case of any virtual intervention that can limit personal contact.

#### 3.1.5. Theme 5: SMS-Based mHealth for Future Pandemics and Epidemics:

Most participants agreed that such interventions can be essential for future public health responses to emerging pathogens.

“*Yeah, I think using SMS-based digital health is the way to go forward for future epidemics…you need a system that allow a direct interaction with people who are having a problem and allow for real-time decision-making… the easiest way to do that is just by exchanging SMS. Otherwise, physical contact might not be possible and other tools say email might not be possible because not everyone has access to internet. But there is high probability that everyone has a phone, or one house member has a phone which they can use for SMS.” [Josh]*

*“In future with regards to fighting epidemics, using [mhealth] and digital health is the way to go I would say” [Marie]*

Vincent commented on the sustainability aspect of SMS-based mHealth interventions and linked it to the context of developing countries.

“*In developing countries like in Africa, I recommend such technologies because it is sustainable approach than any other technologies used for reporting and monitoring”*

This reflects the perceived generalizability of this intervention to LMICs.

## 4. Discussion

### 4.1. Main findings

This study provided insights into the rationale, perspectives, and experiences of KIs in Rwanda towards adopting WelTel intervention in HBC for remote COVID-19 cases and contacts management and support. Participants shared many similar experiences and perspectives. All participants were advocates of the digital-health-centered policies applied in Rwanda for the COVID-19 response.

Rwanda adapted quickly to the increasing demand created by the COVID-19 pandemic through implementing innovative technologies and rolling out the HBC program (19, 24, 27). There is growing evidence that DHIs had a critical role globally in reducing the human and economic impacts of COVID-19 (7, 60). Remote monitoring of COVID-19 cases alleviates strain on low-resourced healthcare systems, protects HCPs, and reduces stigma due to COVID-19 infection (11, 17, 19). However, establishing a system to supervise cases and contacts in home-isolation is critical for effective and safe isolation.

The breadth of reach and timely communication were identified by KIs as major factors to adopting WelTel. This is consistent with literature assessing public health responses to epidemics (7, 61). Studies evaluating the use of DHIs for COVID-19 control recommend the use of mobile technologies to target timely communication (44). However, compared to phone calls and mobile applications, SMS-based mHealth is seen to be more effective at enabling inclusive access to the targeted population, thus increasing breadth of reach (7, 42, 43). In Rwanda, this fact is supported by the relatively high use of basic phones (78%) compared to smartphones (14%), and high rates of SMS use (7, 41, 62). The impact of timely communication can be more effective when remotely monitored cases and contacts can engage in an open-ended manner with HCPs. The 2-way SMS messaging capability of WelTel was identified as another aspect of selecting the tool for HBC. A qualitative study evaluated the experience of veterans with COVID-19 who were remotely monitored using a platform that sends automated check-in SMS messages and share educational information related to COVID-19 and infection prevention and control measures (63). Registered participants can only reply with specific answers to the check-in questions. When asked what was not helpful with the program, they mentioned the inability to express their needs in an open-ended manner (63). A rapid review evaluating the barriers to implementing remote monitoring technologies to support COVID-19 cases recommended the use of technologies with 2-way communication capabilities to enhance case-health provider engagement (64).

The strong political buy-in was deemed by KIs as a critical factor to facilitate WelTel's implementation. Poor political commitment has been presented as a challenge to implementing DHIs as it can impact resource allocation, the establishment of regularity frameworks, and the establishment of appropriate infrastructure. KIs' enthusiasm for adopting DHIs in healthcare is reflected in Rwanda's national policies. Rwanda established the e-Health Steering Committee in 2009 to lead the development of an e-Health department in the Ministry of Health (65). The committee's mandate is to accelerate the development and the implementation of e-Health systems in Rwanda to enable a sustainable healthcare system. Additionally, local strategies targeted the establishment of a competent workforce in digital health. Rwanda is establishing the East African Center for Excellence for Biomedical Engineering and e-Health to train the new generation of digital health experts in the East African Region (66).

KIs perceived the advanced digital infrastructure in Rwanda as an essential facilitator to WelTel adoption. This is consistent with a scoping review of mHealth interventions which emphasized the importance of robust digital infrastructure in facilitating the adoption of mHealth interventions for COVID-19 response (44). Furthermore, the lack of qualified workforce and suboptimal physical/digital infrastructure were presented as barriers to the successful implementation of DHIs for COVID-19 management according to a study in Zimbabwe (42). Accordingly, Rwanda shows strong potential to scaling mHealth tools and a promising future for a digitized healthcare system (24, 27).

Finally, KIs attributed WelTel's ease of use and ease of learning to its user interface. The mHealth Assessment and Planning for Scale Toolkit (MAPS) considers the system's user interface as one criterion for successful mHealth scale up (67). A user-friendly platform facilitates optimal operation of the system by clinical users and real-time decision making (67).

System interoperability is considered an important technical feature of DHIs especially those aimed to be adopted on a national level. The ability of the system to integrate with other systems and exchange data can increase its value and its potential to scale-up (67). As identified by KIs, WelTel was not fully optimized in terms of interoperability and therefore hindered its potential for a full national-wide scale-up. This finding was parallel to a published assessment of WelTel platform (3).

Considering the emergency state created by COVID-19, several countries suspended some data privacy and security regulations to immediately implement DHIs (60). Such approach was possibly adopted globally to accelerate implementation. Although some participants discussed the ability of the platform to auto-logout indicating its ability to protect against unintentional exposure of cases and contacts data, discussion of system-level procedures for data privacy and security, as well as adherence to data standards was lacking. Understanding this aspect of the WelTel implementation is important to identify key technical and regulatory steps taken to protect the privacy and security of cases and contacts data. Furthermore, KIs did not discuss the implications of sharing a single phone in households with multiple members on privacy and anonymity.

Manual data entry and WelTel's inability to conduct phone calls were perceived as barriers to adoption. These were observed to create inefficiencies according to a study in Ireland that measured the effectiveness of a daily automated text-messaging remote active surveillance system of asymptomatic close contacts (68). While the response rate was high (*n* = 10,300; 82%), the study found the absolute number non-respondents to be high (*n* = 2,121; 17%). This 17% created a substantial workload on clinical staff as they had to follow-up with phone calls outside the system (68). Unlike the current study, the percentage of non-respondents was not fully attributed to the local preference for phone calls over texting. However, KIs identified that some cases and contacts preferred phone calls over SMS and indicated the benefit of platforms that enable multimodal comminutions to capture more people. Accordingly, SMS can greatly improve efficiency and reduce the need for phone calls, however, some patients may respond better to phone calls over SMS. Interventions with communication multimodality capabilities can help offload HCP to call just those who need/prefer it. Additionally, the manual data entry of contacts information by clinical staff was considered a resource-intensive system as the number of trained staff increased as the number of contacts increased (68).

The WHO guide on home care of COVID-19 cases and contacts advises public health systems to establish the capacity for communication between HCPs and cases and contacts during the entirety of the home-isolation period (69). Suboptimal supervision, poor infection prevention and control knowledge, and constant fear of the potential to infect house members places an additional burden on the mental health of cases and contacts in home-isoaltion (15, 70). Therefore, addressing these issues could lead to a positive and effective home-isolation. KIs stated that daily monitoring and 2-way SMS communication through WelTel allowed cases and contacts to access reliable information about COVID-19 and infection prevention and control measures and allowed case managers at command posts to closely monitor the evolution of symptoms and epidemiologists to estimate secondary attack rates.

An automated SMS program used to support veterans during COVID-19 found that participants enjoyed being checked on, felt more connected and cared for, and felt less anxious (63). This finding was shared by KIs and accordingly, provides preliminary insights from Rwanda on the impact of SMS-based mHealth on the mental and emotional wellbeing of cases and contacts in home-isolation. Furthermore, the establishment of a channel to connect cases and contacts to a trusted source of information seemed to increase their inclination to continue using according to KIs' experience with WelTel. COVID Watch, a text-messaging system implemented in the US for remote monitoring of COVID-19 cases, enrolled 3,000 cases for automated twice-daily messaging to assess their health status for 14 days (71). Half of the cases in the study asked to extend their enrollment for 21 days. This preference was seen in this study as one KI mentioned the fact that questions about COVID-19 continued post deregistration from the WelTel platform.

Although this study didn't aim to evaluate WelTel's ability to detect cases and reduce secondary attack rates at home, KIs discussed the advantage of the platform in enabling them to ascertain the magnitude of transmission within household settings and prevent further transmission. This is critical given findings of high household secondary attack rates (2.93%) in Rwanda (25). The ability for early case detection and breaking chains of transmission was documented as an advantage of the SMS-based system in Ireland (68).

Urbanization, globalization, and climate change places communities around the world are at greater risk of pandemics (72). This risk is expected to continue and increase over time. The risk of outbreaks of emerging pathogens is greater in developing countries with limited resources and relativity weak public health systems (73). KIs shared their perspective on the usefulness and the applicability of the WelTel intervention to supporting the response to COVID-19 in Rwanda and advocated for its use within emergency management systems in LMICs.

### 4.2. Strengths and limitations

This study brings knowledge and experience about implementing DHIs from a country not commonly presented at the forefront when it comes to digital health. A major strength of this study is the heterogeneity of the sample, which allowed to bring different perspectives and experiences of individuals involved in different levels within Rwanda's national response to COVID-19.

Thematic analysis is known to be a flexible analytical method suitable as it doesn't restrict the researcher to any pragmatic orientation and offers the ability to investigate any research question with great flexibility (57). The researchers past and current involvement with WelTel may introduce bias in data interpretation. The creditability of this research was ensured by constant reflexivity of the researchers' positionality (i.e., identifying researcher characteristics that may influence the study including but not limited to assumptions, values, professional experience, and qualifications and review of stored research transcripts.

Language is a central aspect of qualitative research, and concepts can have different meanings across languages and cultures (74). Participants opted to interview in English versus their local language, and this could have impacted the sophistication of their responses and interpretation of the questions. Thus, loss of meaning potentially occurred from the researcher and the participant side.

Although the researchers ensured rigor in data analysis, the analyses was conducted by one individual. This possibly introduced bias in data interpretation. Furthermore, some interviews were unexpectedly cut short, which may have hindered the ability of the researcher to ask the set of questions per the topic guide. Furthermore, the sample size was largely determined by the availability of KIs and time constrains. However, based on the exploratory nature of this study, we believe that the sample size was sufficient to address the aim of this study. Finally, we acknowledge the potential of recruitment bias introduced due to the sampling method used in this study and the small sample size.

## 5. Conclusion

SMS-based mHealth intervention can provide a sustainable, scalable, and effective approach to monitor and support COVID-19 cases and contacts in home-isolation. However, evidence on this approach is concentrated in high-income settings. Additionally, consideration of local resources, local culture, and access to mobile phones is essential for successful implementation. This study sought to explore the rationale, experiences, and perspectives of KIs in Rwanda regarding the adoption of WelTel for remote monitoring and support of cases and contacts in the HBC program. Whilst the study identified several facilitators to adopting WelTel widely across Rwanda, political commitment to digitizing the public health response in Rwanda and advanced digital infrastructures were seen as major contributors to this implementation. Barriers to implementing WelTel were about technical issues such as sub-optimal interoperability of the platform, and the cases and contacts differential preference towards texting and phone calls. Using WelTel was perceived to enhance the safety and effectiveness of home-isolation through trusted communication, access to information, and data-based decision making. Collaboration between key stakeholders (e.g., governmental, private, and non-governmental sectors) is required to identify solutions for addressing barriers to adopting digital mHealth solutions on a national level.

## Data Availability

Freely available data is presented as anonymized quotes in the results section and in the tables and figures used in this manuscript. In compliance with the university's Research Ethics Board, the raw data supporting the conclusions of this article will be made available by the corresponding author upon request.

## References

[1] WHO. Archived: WHO Timeline—COVID-19. World Health Organization. (2020). Available at: https://www.who.int/news/item/27-04-2020-who-timeline—covid-19 (cited July 17, 2021).

[2] JHU. COVID-19 Map—Johns Hopkins Coronavirus Resource Center. Johns Hopkins University. (2022). Available at: https://coronavirus.jhu.edu/map.html (cited September 8, 2022).

[3] Agarwal S Jalan M Pandya S Ferguson R Moustafa D Ng C Digital Solutions for COVID-19 Response: An assessment of digital tools for rapid scale-up for case management and contact tracing. Report. (2020). p. 1–67. Available at: https://www.jhsph.edu/departments/international-health/news/johns-hopkins-researchers-publish-assessment-of-digital-solutions-for-covid-19-response-in-low-and-middle-income-countries.html (cited July 16, 2021).

[4] WHO. WHO Guideline: Recommendations on Digital Interventions for Health System Strengthening. World Health Organization. (2019). Available at: https://www.ncbi.nlm.nih.gov/books/NBK541902/pdf/Bookshelf_NBK541902.pdf (cited July 17, 2021). 31162915

[5] Ohannessian R Duong TA Odone A. Global telemedicine implementation and integration within health systems to fight the COVID-19 pandemic: a call to action. JMIR Public Health Surveill. (2020) 6(2):1–4. 10.2196/18810 PMC7124951 32238336

[6] Iyengar K Jain VK Vaishya R. Pitfalls in telemedicine consultations in the era of COVID 19 and how to avoid them. Diabetes Metab Syndr. (2020) 14(5):797–9. Available at: http://ovidsp.ovid.com/ovidweb.cgi?T=JS&PAGE=reference&D=med17&NEWS=N&AN=32534432 10.1016/j.dsx.2020.06.007 32534432 PMC7280804

[7] Budd J Miller BS Manning EM Lampos V Zhuang M Edelstein M Digital technologies in the public-health response to COVID-19. Nat Med. (2020) 26(8):1183–92. Available at: https://www.nature.com/articles/s41591-020-1011-4 (cited July 17, 2021). 10.1038/s41591-020-1011-4 32770165

[8] Kondylakis H Katehakis DG Kouroubali A Logothetidis F Triantafyllidis A Kalamaras I COVID-19 Mobile apps: a systematic review of the literature. J Med Internet Res. (2020) 22(12):1–18. 10.2196/23170 PMC7732358 33197234

[9] Robinson L Cotten SR Ono H Quan-Haase A Mesch G Chen W Digital inequalities and why they matter. *Information, Communication & Society*. (2015) 18(5):569–82. 10.1080/1369118X20151012532 Available at: https://www.tandfonline.com/doi/abs/10.1080/1369118X.2015.1012532 (cited July 18, 2021).

[10] Jumelle AKL Ispas I. Ethical issues in digital health. Requir Eng Digital Health. (2015):75–93. 10.1007/978-3-319-09798-5_4. Available at: https://link.springer.com/chapter/10.1007/978-3-319-09798-5_4 (cited July 18, 2021).

[11] Fagherazzi G Goetzinger C Rashid MA Aguayo GA Huiart L. Digital health strategies to fight COVID-19 worldwide: challenges, recommendations, and a call for papers. J Med Internet Res. (2020) 22(6):1–19. 10.2196/19284 PMC7298971 32501804

[12] Brors G Norman CD Norekval TM. Accelerated importance of eHealth literacy in the COVID-19 outbreak and beyond. Eur J Cardiovasc Nurs. (2020) 19(6):458–61. Available at: http://cnu.sagepub.com/content/by/year 10.1177/1474515120941307 32667217 PMC7480020

[13] Mobile Internet Connectivity 2020: Sub-Saharan Africa Factsheet [Internet]. GSMA. (2020). Available at: https://www.gsma.com/r/wp-content/uploads/2020/09/Mobile-Internet-Connectivity-SSA-Fact-Sheet.pdf (cited August 21, 2021).

[14] Bahia Kalvin DA. The State of Mobile Internet Connectivity 2020. (2020). Available at: https://www.gsma.com/r/wp-content/uploads/2020/09/GSMA-State-of-Mobile-Internet-Connectivity-Report-2020.pdf (cited August 21, 2021).

[15] Zhu Y Wang C Dong L Xiao M. Home quarantine or centralized quarantine, which is more conducive to fighting COVID-19 pandemic? Brain Behav Immun. (2020) 87:142. 10.1016/j.bbi.2020.05.009 32387346 PMC7199672

[16] Nam NH Tien PTM van Truong L El-Ramly TA Anh PG Hien NT Early centralized isolation strategy for all confirmed cases of COVID-19 remains a core intervention to disrupt the pandemic spreading significantly. PLoS One. (2021) 16(7):e0254012. 10.1371/journal.pone.0254012. Available at: https://journals.plos.org/plosone/article?id=10.1371/journal.pone.0254012 (cited August 7, 2021). 34264966 PMC8282022

[17] Ilesanmi Olayinka S Afolabi Aanuoluwapo A. A scope review on home-based care practices for COVID-19: what Nigeria can learn from other countries – ibom medical journal. Ibom Med J. (2020) 14(1):1–9. Available at: https://ibommedicaljournal.org/index.php/imjhome/article/view/83 (cited August 7, 2021).

[18] Rwanda Biomedical Center. Home Based Isolation and Care Guidelines for Patients with COVID-19. Kigali. (2020). Available at: https://rbc.gov.rw/fileadmin/user_upload/annoucement/Guidelines%20for%20home-based%20care_COVID-19.pdf (cited 2021 Jul 23, 2021).

[19] XinhuaNet. Rwanda lowers costs of fighting COVID-19 through home-based care: official—Xinhua | English.news.cn. XinhuaNet. (2020). Available at: http://www.xinhuanet.com/english/2020-10/03/c_139416538.htm (cited July 23, 2021).

[20] Dickens BL Koo JR Wilder-Smith A Cook AR. Institutional, not home-based, isolation could contain the COVID-19 outbreak. Lancet. (2020) 395(10236):1541. 10.1016/S0140-6736(20)31016-3 32423581 PMC7190294

[21] Worldometer. Rwanda Population. Worldometer. (2022). Available at: https://www.worldometers.info/world-population/rwanda-population/ (cited September 18, 2022).

[22] Government of Rwanda: Administrative structure. Available at: https://www.gov.rw/government/administrative-structure (cited September 18, 2021).

[23] World Population Review. Rwanda Population 2021 (Demographics, Maps, Graphs). (2021). Available at: https://worldpopulationreview.com/countries/rwanda-population (cited July 16, 2021).

[24] Karim N Jing L Lee JA Kharel R Lubetkin D Clancy CM Lessons learned from Rwanda: innovative strategies for prevention and containment of COVID-19. Ann Glob Health. (2021) 87(1):1–9. 10.5334/aogh.3108 33665145 PMC7908927

[25] Semakula M Niragire F Umutoni A Nsanzimana S Ndahindwa V Rwagasore E The secondary transmission pattern of COVID-19 based on contact tracing in Rwanda. BMJ Glob Health. (2021) 6(6):e004885. Available at: https://gh.bmj.com/content/6/6/e004885 (cited July 22, 2021). 10.1136/bmjgh-2020-004885 34103325 PMC8189754

[26] WHO. First Case of COVID-19 confirmed in Rwanda | WHO | Regional Office for Africa. WHO. (2020). Available at: https://www.afro.who.int/news/first-case-covid-19-confirmed-rwanda (cited July 22, 2021).

[27] COVID-19 in Rwanda: A country’s response | WHO | Regional Office for Africa. (2020). Available at: https://www.afro.who.int/news/covid-19-rwanda-countrys-response (cited July 15, 2021).

[28] Hale T Angrist N Goldszmidt R Kira B Petherick A Phillips T A global panel database of pandemic policies (Oxford COVID-19 government response tracker). Nat Hum Behav. (2021) 5(4):529–38. 10.1038/s41562-021-01079-8 33686204

[29] Rwanda: 95% of COVID-19 patients being treated at home. Available at: https://www.aa.com.tr/en/africa/rwanda-95-of-covid-19-patients-being-treated-at-home/2122611 (cited July 16, 2021).

[30] Rwanda Biomedical Center. Home Based Care. Available at: https://www.rbc.gov.rw/index.php?id=760 (cited July 16, 2021).

[31] CNBC. Delta variant: Africa suffers worst surge in Covid cases officials brace for third wave. CNBC. (2021). Available at: https://www.cnbc.com/2021/07/08/delta-variant-africa-suffers-worst-surge-in-covid-cases-officials-brace-for-third-wave.html (cited July 25, 2021).

[32] RBC. COVID19 Weekly Epidemiological Bulletin. Rwanda Biomedical Center. (2021). Available at: https://rbc.gov.rw/fileadmin/user_upload/annoucement/EPI%20WEEK%2037-%20From%20to%2013th%20to%2019th%20September,2021%20.pdf (cited September 25, 2021).

[33] Rwanda Biomedical Center. Rwanda COVID-19 Dashboard. RBC. (2021). Available at: https://www.rbc.gov.rw/index.php?id=707&L=0 (cited July 25, 2021).

[34] Rwanda Biomedical Center. New Cases per District. RBC. (2021). Available at: https://www.rbc.gov.rw/fileadmin/user_upload/annoucement/new%20cases%20per%20district%2023%2007%202021.jpg (cited July 25, 2021).

[35] Rwanda Biomedical Center. COVID19 Weekly Epidemiological Bulletin. Rwanda Biomedical Center. (2022). Available at: https://rbc.gov.rw/fileadmin/user_upload/annoucement/EPIWEEK35From29thAugustto4thSeptember,2022.pdf (cited September 8, 2022).

[36] Nachega JB Leisegang R Kallay O Mills EJ Zumla A Lester RT. Mobile health technology for enhancing the COVID-19 response in Africa: a potential game changer? Am J Trop Med Hyg. (2020) 103(1):3–5. Available at: https://www.ajtmh.org/view/journals/tpmd/103/1/article-p3.xml (cited July 15, 2021). 10.4269/ajtmh.20-0506 32476643 PMC7356462

[37] WelTel. Available from: https://www.weltelhealth.com/Home (cited July 16, 2021).

[38] Binyam SK Bardosh KL Murray M Fitzgerald M Cook V Poureslami I Identifying barriers and facilitators of 13 mHealth projects in North America and Africa: protocol for a 5-year implementation science study. JMIR Res Protoc. (2018) 7(7):e162. https://www.researchprotocols.org/2018/7/e162 (cited July 16, 2021). 10.2196/resprot.9633 29970360 PMC6053607

[39] Lester RT. Ask, don’t tell — mobile phones to improve HIV care. N Engl J Med. (2013) 369(19):1867–8. 10.1056/NEJMc1310509 Available at: https://www.nejm.org/doi/full/10.1056/NEJMc1310509 (cited July 16, 2021). 24195570

[40] Foundation for Innovative New Diagnosis. Use of Digital Tools to Strengthen Covid-19 Management, Rwanda case study. (2021). Available at: https://www.finddx.org/wp-content/uploads/2021/05/FIND_Digital-Health-Report_RWANDA_v1.pdf

[41] RURA. STATISTICS REPORT FOR TELECOM, MEDIA AND BROADCASTING SECTOR AS OF THE SECOND QUARTER OF THE YEAR 2020. Kigali. (2020). Available at: https://rura.rw/fileadmin/Documents/ICT/statistics/ICT_and_Telecom_Statistics_report_as_of_June_2020.pdf (cited August 21, 2021).

[42] Chidhau S Mutizwa B Muzama TR. The impact of the digital health interventions in curbing COVID-19 in Zimbabwe. Int J Clin Invent Med Sci. (2021) 3(1):40–52. Available at: https://lamintang.org/journal/index.php/ijcims/article/view/203 (cited September 11, 2021). 10.36079/lamintang.ijcims-0301.203

[43] Loubet P Czeschan C Sintes M Sotto A Laureillard D. Use of short message service in at-home COVID-19 patient management. BMC Med (2020) 18(1):1–4. 10.1186/s12916-020-01863-9. Available at: https://bmcmedicine.biomedcentral.com/articles/10.1186/s12916-020-01863-9 (cited September 11, 2021). 33323098 PMC7738241

[44] Asadzadeh A Kalankesh LR. A scope of mobile health solutions in COVID-19 pandemics. Inform Med Unlocked. (2021) 23:100558. 10.1016/j.imu.2021.100558 33842688 PMC8019236

[45] Richard L Sabin N Chantal B Gabrielle S Justine U el Joueidi S Implementing an innovative evidence-based Mobile health (mHealth) intervention to improve engagement and adherence to HIV prevention and care services in Rwanda. Kigali: international conference on AIDS and STIs in Africa 2019 (2019). p. 1–360. Available at: https://saafrica.org/new/wp-content/uploads/2020/02/ICASA-2019-Abstract-Book-online-version.pdf (cited July 16, 2021).

[46] O’Brien BC Harris IB Beckman TJ Reed DA Cook DA. Standards for reporting qualitative research: a synthesis of recommendations. Acad Med. (2014) 89(9):1245–51. Available at: https://journals.lww.com/academicmedicine/Fulltext/2014/09000/Standards_for_Reporting_Qualitative_Research__A.21.aspx (cited July 25, 2021). 10.1097/ACM.0000000000000388 24979285

[47] Video Conferencing, Cloud Phone, Webinars, Chat, Virtual Events | Zoom. Available at: https://zoom.us/ (cited September 17, 2021).

[48] el Joueidi S Bardosh K Musoke R Moslim MA Gourlay K Macmullin A Evaluation of the Implementation Process of the Mobile Health Platform “WelTel” in Six Sites in East Africa and Canada Using the Modified Consolidated Framework for Implementation Research (mCFIR). (2020). Available at: https://www.researchsquare.com (cited August 1, 2021). 10.1186/s12911-021-01644-1 PMC8546747 34702229

[49] Bardosh KL Murray M Khaemba AM Smillie K Lester R. Operationalizing mHealth to improve patient care: a qualitative implementation science evaluation of the WelTel texting intervention in Canada and Kenya. Global Health. (2017) 13(1):1–15. Available at: https://globalizationandhealth.biomedcentral.com/articles/10.1186/s12992-017-0311-z (cited Aug 1, 2021). 10.1186/s12992-017-0311-z 29208026 PMC5717811

[50] Keith RE Crosson JC O’Malley AS Cromp D Taylor EF. Using the consolidated framework for implementation research (CFIR) to produce actionable findings: a rapid-cycle evaluation approach to improving implementation. Implement Sci. (2017) 12(1):1–12. Available at: https://implementationscience.biomedcentral.com/articles/10.1186/s13012-017-0550-7 (cited August 1, 2021). 10.1186/s13012-017-0550-7 28187747 PMC5303301

[51] mHealth Research Group | Infectious Diseases [Internet]. University of British Columbia. Available at: https://id.med.ubc.ca/education/infectious-diseases-research-education/research/mhealth/ (cited September 25, 2021).

[52] Damschroder LJ Aron DC Keith RE Kirsh SR Alexander JA Lowery JC. Fostering implementation of health services research findings into practice: a consolidated framework for advancing implementation science. Implement Sci. (2009) 4(1):1–15. Available at: https://implementationscience.biomedcentral.com/articles/10.1186/1748-5908-4-50 (cited August 21, 2021). 10.1186/1748-5908-4-50 19664226 PMC2736161

[53] Means AR Kemp CG Gwayi-Chore MC Gimbel S Soi C Sherr K Evaluating and optimizing the consolidated framework for implementation research (CFIR) for use in low- and middle-income countries: a systematic review. Implement Sci. (2020) 15(1):1–19. Available at: https://implementationscience.biomedcentral.com/articles/10.1186/s13012-020-0977-0 (cited August 21, 2021). 10.1186/s13012-020-0977-0 32164692 PMC7069199

[54] Alison M Chantler T. Principles of social research pdf (by mary alison durand, tracey chantler) read online. Maidenhead, England: Open University Press (2014). 185 p. Available at: https://search.ebscohost.com/login.aspx?direct=true&AuthType=cookie,ip,shib&db=nlebk&AN=821729&site=ehost-live (cited July 28, 2021).

[55] Braun V Clarke V. Using thematic analysis in psychology. Qual Res Psychol. (2006) 3(2):77–101. Available from: http://www.tandfonline.com/loi/uqrp20 (cited July 17, 2021). 10.1191/1478088706qp063oa

[56] Fereday J Adelaide N Australia S Eimear Muir-Cochrane A. Demonstrating rigor using thematic analysis: A hybrid approach of inductive and deductive coding and theme development. International Journal of Qualitative Methods (2006) 5(1):80–92. 10.1177/160940690600500107

[57] Kiger ME Varpio L. Medical Teacher Thematic analysis of qualitative data: AMEE Guide No. 131 Thematic analysis of qualitative data: AMEE Guide No. 131. Available at: 10.1080/0142159X.2020.1755030 (cited September 17, 2021). 32356468

[58] Latest NVivo Version. NVIVO. (2020). Available at: https://www.qsrinternational.com/nvivo-qualitative-data-analysis-software/upgrade-nvivo (cited September 17, 2021).

[59] Berger R. Now I see it, now I don’t: researcher’s position and reflexivity in qualitative research. Qual Res. (2015) 15(2):219–34. 10.1177/1468794112468475

[60] Mahmood S Hasan K Carras MC Labrique A. Global preparedness against COVID-19: we must leverage the power of digital health. JMIR Public Health Surveill. (2020) 6(2):e18980. Available at: https://publichealth.jmir.org/2020/2/e18980 (cited September 15, 2021). 10.2196/18980 32297868 PMC7164944

[61] Gong N Jin X Liao J Li Y Zhang M Cheng Y Authorized, clear and timely communication of risk to guide public perception and action: lessons of COVID-19 from China. BMC Public Health. (2021) 21(1):1–8. Available at: https://bmcpublichealth.biomedcentral.com/articles/10.1186/s12889-021-11103-1 (cited September 11, 2021). 10.1186/s12889-020-10013-y 34384378 PMC8358541

[62] Tasamba J. Rwanda aims to collect 1M smartphones for poor families. (2020). Available at: https://www.aa.com.tr/en/africa/rwanda-aims-to-collect-1m-smartphones-for-poor-families/1704126 (cited September 11, 2021).

[63] Saleem JJ Read JM Loehr BM Frisbee KL Wilck NR Murphy JJ Veterans’ response to an automated text messaging protocol during the COVID-19 pandemic. J Am Med Inform Assoc. (2020) 27(8):1300–5. Available at: https://pubmed.ncbi.nlm.nih.gov/32470974/ (cited September 11, 2021). 10.1093/jamia/ocaa122 32470974 PMC7313999

[64] Houlding E Mate KKV Engler K Ortiz-Paredes D Pomey MP Cox J Barriers to use of remote monitoring technologies used to support patients with COVID-19: rapid review. JMIR Mhealth Uhealth. (2021) 9(4):e24743. Available at: https://mhealth.jmir.org/2021/4/e24743 (cited September 15, 2021). 10.2196/24743 33769943 PMC8059785

[65] Richard G. The National E-Health Strategic Plan 2009–2013. Kigali. (2009). Available at: https://www.who.int/goe/policies/Rwanda_national_ehealth_strategy_2009-2013.pdf?ua=1 (cited September 12, 2021).

[66] National Strategy For Health Professions Development 2020–2030. Rwanda Biomedical Center. (2020). p. 1–187. Available at: https://www.rbc.gov.rw/fileadmin/user_upload/strategy/RWANDA%20National%20Strategy%20for%20Health%20Professions%20Development%20%28NSHPD%202020-2030%29.pdf (cited September 12, 2021).

[67] World Health Organization. The MAPS Toolkit mHealth Assessment and Planning for Scale Global mHealth Initiative WHO Library Cataloguing-in-Publication Data The MAPS toolkit: mHealth assessment and planning for scale. Geneva. (2015). Available at: https://apps.who.int/iris/bitstream/handle/10665/185238/9789241509510_eng.pdf?sequence=1&isAllowed=y (cited September 15, 2021).

[68] Bambury N Condon R Crompton J Sheahan A Barrett PM Kelly L. Measuring the effectiveness of an automated text messaging active surveillance system for COVID-19 in the south of Ireland, march to April 2020. Eurosurveillance. (2020) 25(23):2000972. 10.2807/1560-7917.ES.2020.25.23.2000972. Available at: https://www.eurosurveillance.org/docserver/fulltext/eurosurveillance/25/23/eurosurv-25-23-3.pdf?expires=1595677264&id=id&accname=guest&checksum=360592B429E12EA055B31F387389AB2E. 32553064 PMC7403640

[69] World Health Organization. Home care for patients with suspected or confirmed COVID-19 and management of their contacts. Geneva. (2020). Available at: https://www.who.int/publications/i/item/home-care-for-patients-with-suspected-novel-coronavirus-(ncov)-infection-presenting-with-mild-symptoms-and-management-of-contacts (cited September 16, 2021).

[70] Mohamed AE Yousef AM. Depressive, anxiety, and post-traumatic stress symptoms affecting hospitalized and home-isolated COVID-19 patients: a comparative cross-sectional study. Middle East Current Psychiatry. (2021) 28(1):1–12. Available at: https://mecp.springeropen.com/articles/10.1186/s43045-021-00105-9 (cited August 6, 2021). 10.1186/s43045-021-00105-9

[71] Morgan AU Balachandran M Do D Lam D Parambath A Chaiyachati KH Remote Monitoring of Patients with Covid-19: Design, implementation, and outcomes of the first 3,000 patients in COVID Watch. Catalyst non-issue content. (2020). Available at: https://catalyst.nejm.org/doi/full/10.1056/CAT.20.0342 (cited September 12, 2021).

[72] Madhav N Oppenheim B Gallivan M Mulembakani P Rubin E Wolfe N. Pandemics: risks, impacts, and mitigation. In: Patel V, Chisholm D, Dua T, Laxminarayan R, Medina-Mora ME, editors. Disease control priorities, 3rd ed. Vol. 9: Improving Health and Reducing Poverty. Washington: International Bank for Reconstruction and Development (2017). p. 315–45. Available at: https://www.ncbi.nlm.nih.gov/books/NBK525302/ (cited September 17, 2021).

[73] Why It Matters: The Pandemic Threat. Division of Global Health Protection. Global Health. CDC. Available at: https://www.cdc.gov/globalhealth/healthprotection/fieldupdates/winter-2017/why-it-matters.html (cited September 17, 2021).

[74] van Nes F Abma T Jonsson H Deeg D. Language differences in qualitative research: is meaning lost in translation? Eur J Ageing. (2010) 7(4):313. 10.1007/s10433-010-0168-y 21212820 PMC2995873

