# Supplementary material for: SMS-based digital health intervention in Rwanda's home-based care program for remote management of COVID-19 cases and contacts: A qualitative study of sustainability and scalability
Source: Front Digit Health. 2023 Jan 9;4:1071790. doi: 10.3389/fdgth.2022.1071790 (PMC9879010; doi:10.3389/fdgth.2022.1071790)
Supplement: Supplementary file 1 [file Datasheet1.docx]

Supplementary Material

# Supplementary Data

Supplementary Material should be uploaded separately on submission. Please include any supplementary data, figures and/or tables. All supplementary files are deposited to FigShare for permanent storage and receive a DOI.

Supplementary material is not typeset so please ensure that all information is clearly presented, the appropriate caption is included in the file and not in the manuscript, and that the style conforms to the rest of the article. To avoid discrepancies between the published article and the supplementary material, please do not add the title, author list, affiliations or correspondence in the supplementary files.

# Supplementary Figures and Tables

- 1. All the reproduced figures are published under a Creative Commons CC-BY Attribution 4.0 International (CC BY 4.0). Therefore, the author is free to copy and redistribute the material in any medium or format


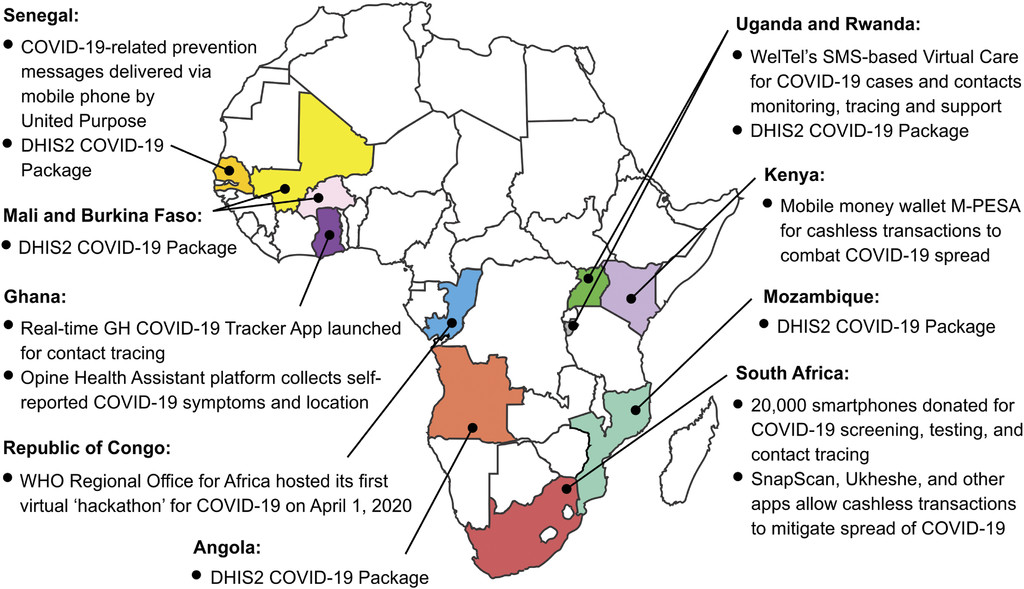


Figure 3. Implementation of WelTel in East Africa: WelTel was adopted in Rwanda and Uganda to support local efforts towards the control of COVID-19. This figure was reproduced from Nachega.^36^


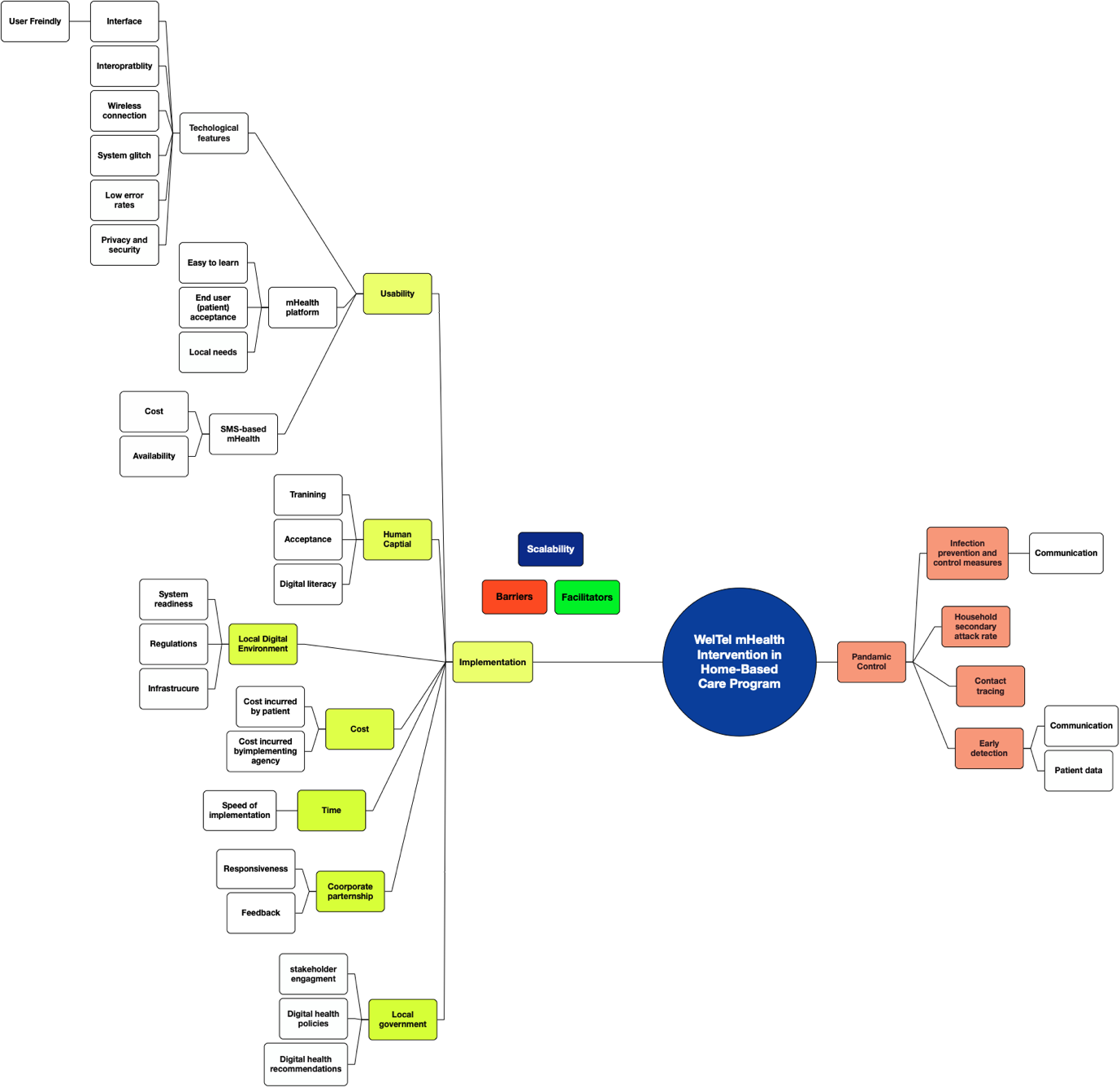


Figure 4. Pre-coding Map

Table 2 mCFIR Framework: The mCFIR consists of 25 constructs under 5 major domains including: (1) Intervention Characteristics; (2) Outer Setting - Outside the implementer team; (3) Inner Setting - Within the Implementer Team; (4A) End-User Characteristics - Health Care Providers; (4B) End-User Characteristics – Patients; (5) Implementation Process. This table was reproduced from Joueidi.^48^

| **Domain Title** | **Construct Descriptive** | **Construct Title** |
| --- | --- | --- |
| **Domain 1 - Intervention Characteristics** | **Construct 1**  How well does the intervention perform in its intended setting? | Performance Fidelity |
| **Domain 1 - Intervention Characteristics** | **Construct 2**  How adaptable is this intervention to meeting local needs? | Adaptability |
| **Domain 1 - Intervention Characteristics** | **Construct 3**  How user-friendly is the service of this intervention? | User-friendliness |
| **Domain 1 - Intervention Characteristics** | **Construct 4** How advantageous is this intervention over alternative solutions that are already in place? | Comparative advantage |
| **Domain 1 - Intervention Characteristics** | **Construct 5**  How acceptable are the costs of the intervention to the implementer and users? | Affordability |
| **Domain 2 - Outer Setting** | **Construct 6**  How well connected is the project implementer team with outer setting stakeholders? | Stakeholder Engagement |
| **Domain 2 - Outer Setting** | **Construct 7**  Do external stakeholders or competing organizations support the implementation of the intervention? | External support |
| **Domain 2 - Outer Setting** | **Construct 8**  How do external strategies contribute to spread this intervention? | Scale-up support |
| **Domain 3 - Inner Setting** | **Construct 9**  How strong is the networking and communication within the target organization? | Internal communication |
| **Domain 3 - Inner Setting** | **Construct 10**  Is use of the intervention welcomed, encouraged, and supported within the inner setting? | Acceptance |
| **Domain 3 - Inner Setting** | **Construct 11**  How well is the infrastructure and logistic support provided to the intervention by your organization? | Organizational Support |
| **Domain 4A - End-User Characteristics, HCP** | **Construct 12**  The health care providers believe that the intervention improves health outcomes. | Benefit Perception |
| **Domain 4A - End-User Characteristics, HCP** | **Construct 13**  The health care providers feel well-trained and confident while using the intervention. | [HCP] Training |
| **Domain 4A - End-User Characteristics, HCP** | **Construct 14**  The health care providers feel secure with the level of privacy offered by the intervention. | [HCP] Privacy |
| **Domain 4B - End-User Characteristics, Patients** | **Construct 15**  Patients believe the intervention improves their health outcomes compared to the current practice. | Benefit Perception |
| **Domain 4B - End-User Characteristics, Patients** | **Construct 16**  Patients feel confident in their own ability to use the intervention. | [Pt.] Training |
| **Domain 4B - End-User Characteristics, Patients** | **Construct 17**  Patients have adequate access to the service the intervention provides. | Accessibility |
| **Domain 4B - End-User Characteristics, Patients** | **Construct 18**  Patients feel secure with the level of privacy offered by the intervention. | [Pt.] Privacy |
| **Domain 4B - End-User Characteristics, Patients** | **Construct 19**  Patients understand the language used in the intervention. | Language |
| **Domain 5 - Implementation Process** | **Construct 20**  How adequate has attention been given to planning the implementation towards the set goal? | Intervention planning |
| **Domain 5 - Implementation Process** | **Construct 21**  How well are the stakeholders engaged during the implementation? | Stakeholder engagement |
| **Domain 5 - Implementation Process** | **Construct 22**  How well has the intervention been carried out according to plan? | Execution |
| **Domain 5 - Implementation Process** | **Construct 23**  Is time dedicated for reflection or debriefing about the implementation before, during and after the implementation process? | Evaluation |
| **Goal Attainment Scale** | **Construct 24**  How well are you achieving your implementation goals? | Goal attainment |
| **Impact Assessment** | **Construct 25**  How well are you achieving your key outcomes? | Outcome Assessment |
